# Targeting the autophagy-lysosomal pathway in Huntington disease: a pharmacological perspective

**DOI:** 10.3389/fnagi.2023.1175598

**Published:** 2023-05-25

**Authors:** Junsheng Yang, Chaoyue Zhang

**Affiliations:** Collaborative Innovation Center of Yangtze River Delta Region Green Pharmaceuticals, College of Pharmaceutical Sciences, Zhejiang University of Technology, Hangzhou, China

**Keywords:** autophagy-lysosomal pathway, Huntington disease, mHTT, aggrephagy, targeted protein degradation

## Abstract

The autophagy-lysosomal pathway (ALP) is the major biological pathway responsible for clearing intracellular protein aggregates, therefore a promising target for treating diseases featuring the accumulation of aggregation-prone proteins, such as Huntington disease (HD). However, accumulating evidence indicated that targeting ALP to treat HD is pharmacologically challenging due to the complexity of autophagy and the autophagy defects in HD cells. Here in this mini-review, we summarized the current challenges in targeting ALP in HD and discussed a number of latest findings on aggrephagy and targeted protein degradation, which we believe will provide potential new targets and new strategies for treating HD via ALP.

## Introduction

Huntington disease (HD) is a monogenic neurodegenerative disease caused by a dominantly inherited CAG expansion in exon 1 of the huntingtin gene (*HTT*) and the resulting misfolding- and aggregate-prone mutant hungtingtin protein (mHTT) (Bates et al., 2015). Although it still remains controversial whether the mHTT aggregates found in HD patients’ brains are toxic or rather a result of cell defense mechanisms against more toxic mHTT oligomers, lowering mHTT has been shown to alleviate HD pathology (Jimenez-Sanchez et al., 2017; Tabrizi et al., 2020). Tremendous efforts have thus been put into mHTT-lowering strategies such as gene therapy to reduce mHTT production or boosting protein quality control mechanisms to enhance mHTT degradation, for their potential clinical benefits.

The ubiquitin-proteasome system (UPS) and autophagy lysosomal pathway (ALP) are the two major mechanisms used by mammalian cells for protein degradation. While UPS is mainly responsible for the degradation of soluble proteins, ALP degrades a wider spectrum of substrates including protein aggregates. Accumulating evidence indicate that boosting ALP can lower mHTT and improve cell survival in both cellular and animal models of HD (Djajadikerta et al., 2020), making ALP a promising pharmaceutical target for HD. However, to effectively and selectively lower mHTT via ALP up-regulation is still a challenging task for reasons discussed below.

## Targeting ALP in HD: challenges

Autophagy-lysosomal pathway is compromised in HD at multiple levels. A recent study comparing the gene expression profiles of striatal neurons directly reprogrammed from fibroblasts of HD patients with healthy controls revealed a down-regulation of genes enriched in ALP, consistent with a decreased autophagy activity measured by functional assays (Oh et al., 2022). In line with this, another study indicated that transcription factor EB (TFEB), the master regulator of autophagy and lysosome biogenesis, contains a prion-like domain (PrLD) that can mediate its co-aggregation with mHTT both *in vitro* and *in vivo* (Yang et al., 2023). This co-aggregation disrupted TFEB activation in a cell-based reporting system. Interestingly, the PrLD is unique to TFEB but not found in transcription factor E3 (TFE3), another transcription factor of the microphthalmia family (MiT) that can also regulate ALP activation. There are also *in vivo* studies in HD mice models indicating an overall activation of ALP by overexpressing TFEB lead to ER stress and reactive astrogliosis (Vodicka et al., 2016) or failed to alleviate HD disease phenotypes (Brattås et al., 2021). These results suggest there is an overall decreased production of ALP components in HD cells and targeting TFEB to restore that might be problematic. Alternatively, it is worth testing whether specifically activating TFE3 in HD can be beneficial.

A number of studies also showed that the ALP in HD cells is not fully functional. The finding of empty autophagosomes in HD cells is indicative of a defect in either cargo recognition or cargo engulfing (Martinez-Vicente et al., 2010; Yang et al., 2021). Autophagosome trafficking defects (Wong and Holzbaur, 2014) and impaired autophagosome-lysosome fusion (Pircs et al., 2018) have also been reported in mHTT-expressing neurons. In selective autophagy, wild type hungtingtin protein (WtHTT) was found to play a role as a scaffold (Rui et al., 2015), while mHTT appeared to impair LC3-p62 interaction and lead to defected mitophagy (Franco-Iborra et al., 2021).

Taken together, an overall elevation of ALP in HD could be both challenging and yet ineffective. On the other hand, with the advancing understandings of the molecular mechanisms of ALP, more potential targets and novel strategies are emerging.

## Targeting ALP in HD: new targets

Besides boosting the overall ALP activity, an alternative possibility to lower mHTT aggregates via ALP is to increase aggrephagy, a form of selective autophagy that specifically degrades intracellular aggregates.

Unlike bulk autophagy, selective autophagy is capable of selectively recognizing and targeting substrates such as lipids (lipophagy), ER (ER-phagy), mitochondria (mitophagy), and aggregates (aggrephagy), with the assistance of a range of receptors that can recognize their specific cargos and bridge them to LC3 via their LC3-interacting region (LIR) (Gatica et al., 2018; Lamark and Johansen, 2021). Since the first recognition of p62/SQSTM1 as a selective autophagy receptor that can facilitate the clearance of mHTT aggregates (Bjørkøy et al., 2005), a few more receptors for aggrephagy have been identified, among which optineurin (OPTN) (Korac et al., 2013), the CUET protein Tollip (Lu et al., 2014), and TAX1BP1 (Sarraf et al., 2020) were reported to be able to mediate the degradation of mHTT aggregates. Mechanism-wise, both Tollip and TAX1BP1 work dependent on the ubiquitination of the cargoes. OPTN was first thought to recognize aggregates and mediate aggrephagy via its C-terminal coiled-coil domain independent of its ubiquitin binding domain (UbBD) (Korac et al., 2013). But a later study indicated that the aggrephagy receptor function of OPTN is still ubiquitin-dependent (Shen et al., 2015). The authors revealed that the UbBD-mutated OPTN can interact with WT OPTN to form WT-mutant hybrids and therefore get indirectly associated with ubiquitinated aggregates.

In 2022, Ma et al. (2022) reported the chaperonin TRiC subunit CCT2 as a new receptor that can regulate the selective autophagy of aggregation-prone proteins including mHTT. The aggrephagy receptor function of CCT2 is independent of TRiC. Interestingly, CCT2 manifests several unique properties compared with other aggrephagy receptors. First, CCT2 interacts with ATG8 family members not through a canonical LC3-interacting region (LIR), but via a motif termed V-LC3-interacting region (VLIR), which contains two adjacent hydrophobic regions that both include a V residue. Second, CCT2 targets mHTT aggregates to autophagosomes independent of ubiquitin. Third, CCT2 appeared to be specific to aggrephagy. Fourth and most interestingly, CCT2 showed a preference on aggregates with little liquidity when tested on an established fused in sarcoma (FUS) liquid-to-solid transition model. This is probably due to CCT2’s preferred binding with solid aggregates. Considering mHTT also undergo a liquid-to-solid phase transition over time (Peskett et al., 2018; Posey et al., 2018; Aktar et al., 2019), the CCT2-mediated degradation of solid aggregates could be especially noteworthy for treating HD cells.

Taken together, the discovering of aggrephagy receptors made it possible to target more precisely on a subset of the autophagy machinery to more specifically degrade pathological mHTT species. The newly identified aggrephagy receptor CCT2 is worth extra attention because of its unique specificity with solid aggregates. The binding affinity of CCT2 with mHTT species of different liquidity is especially worth exploring, considering mHTT can also go through liquid-to-solid phase transition (Peskett et al., 2018; Yang and Yang, 2020). The mHTT lowering capacity of overexpressed CCT2 both *in vitro* and *in vivo* is also encouraging (Ma et al., 2022). However, to pharmaceutically up-regulate mHTT-specific aggrephagy or overall aggrephagy still remains challenging. Post-translational modifications that modulate the target recognition by aggrephagy receptors, such as the phosphorylation of OPTN by TANK1 binding kinase 1 (TBK1) (Korac et al., 2013), could be potentially “druggable.” But to find such potential pharmacological targets, a much more thorough understanding of the mechanisms how aggrephagy receptors specifically recognize and target aggregates for degradation will be needed.

## Targeting ALP in HD: new strategies

Besides the aforementioned studies that shed light on the molecular biology of mHTT degradation, exciting works have also been done using an alternative strategy termed targeted protein degradation (TPD), which utilizes synthetic hetero-bifunctional molecules to bridge the protein targets with the intracellular degradation machineries such as UPS and ALP (Békés et al., 2022).

The first developed TPD concept is the PROteolysis TArgeting Chimeras (PROTACs), in which molecules were designed to link the protein of interest with a ubiquitin E3 ligase (Sakamoto et al., 2001). Although the idea of PROTACs can be traced back to more than 20 years ago, it is until recently two studies reported that PROTACs targeting aggregate-prone proteins to the ubiquitin E3 ligase cIAP can reduce mHTT in fibroblasts from HD patients (Tomoshige et al., 2017, 2018). Surprisingly, these PROTACs, which were originally designed to target aggregate-prone mHTT, also reduced soluble mHTT and WtHTT through unknown mechanisms.

With a similar idea, Li et al. (2019) developed a new TPD strategy termed AuTophagy-TEthering Compounds (ATTECs) that can link LC3 and mHTT. To find ATTECs that specifically promote the degradation of mHTT but not WtHTT, the authors combined small-molecule-microarray based screens and counter-screens and successfully discovered four compounds that bind both LC3 and mHTT. In efficacy studies, all of the four compounds were able to reduce mHTT in HD mouse neurons; three of them could also reduce mHTT in cortices of HD mice when administrated by intracerebroventricular injections. Moreover, two compounds were capable of penetrating the blood brain barrier (BBB) and lowering cortical and striatal mHTT in HD mice when dosed via intraperitoneal injection.

Beside ATTECs, more TPD technologies that aim to hijack ALP are being developed to degrade diverse substrates [see (Ding et al., 2022) for a timely and extensive review]. The possibility of applying them to lower mHTT surely is worth exploring.

## Concluding remarks

**FIGURE 1:**
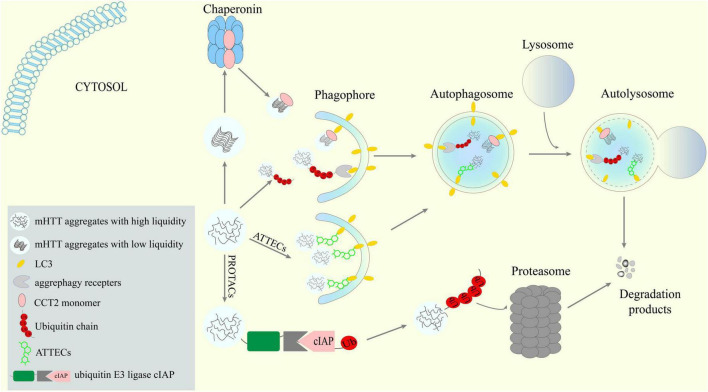
Potential new targets and strategies to lower mHTT aggregates via ALP. Receptors-mediated aggrephagy, especially the newly discovered CCT2-mediated degradation of solid aggregates, are potential targets for mHTT clearance. New targeted protein degradation (TPD) techniques such as AuTophagy-TEthering Compounds (ATTECs) may provide means to hijack the ALP to lower mHTT.

Although lowering mHTT via ALP is an intriguing idea, targeting ALP to treat HD is pharmacologically challenging due to the complexity of autophagy *per se*, the compromised autophagy function in HD cells and the lack of druggable targets. Recent findings discussed above have not only added more knowledge in the molecular mechanisms of mHTT degradation by the ALP, but also provided successful proof-of-concept studies supporting novel TPD technologies (see Figure 1 for a graphic summary). With the combination of these new knowledge and technologies, more efficient and specific approaches to pharmaceutically lower mHTT can be expected.

## Author contributions

JY and CZ discussed and wrote the manuscript. Both authors contributed to the article and approved the submitted version.
